# O-Fucosylation of DLL3 Is Required for Its Function during Somitogenesis

**DOI:** 10.1371/journal.pone.0123776

**Published:** 2015-04-09

**Authors:** Katrin Serth, Karin Schuster-Gossler, Elisabeth Kremmer, Birte Hansen, Britta Marohn-Köhn, Achim Gossler

**Affiliations:** 1 Institut für Molekularbiologie OE5250, Medizinische Hochschule Hannover, Carl-Neuberg-Str.1, 30625, Hannover, Germany; 2 Helmholtz Zentrum München, German Research Center for Environmental Health, Institute of Molecular Immunology, Marchioninistrasse 25, 81377, Munich, Germany; Instituto de Medicina Molecular, PORTUGAL

## Abstract

Delta-like 3 (DLL3) is a member of the DSL family of Notch ligands in amniotes. In contrast to DLL1 and DLL4, the other Delta-like proteins in the mouse, DLL3 does not bind in trans to Notch and does not activate the receptor, but shows cis-interaction and cis-inhibitory properties on Notch signaling in vitro. Loss of the DSL protein DLL3 in the mouse results in severe somite patterning defects, which are virtually indistinguishable from the defects in mice that lack lunatic fringe (LFNG), a glycosyltransferase involved in modifying Notch signaling. Like LFNG, DLL3 is located within the trans-Golgi, however, its biochemical function is still unclear. Here, we show that i) both proteins interact, ii) epidermal growth factor like repeats 2 and 5 of DLL3 are O-fucosylated at consensus sites for POFUT1, and iii) further modified by FNG proteins in vitro. Embryos double homozygous for null mutations in *Dll3* and *Lfng* are phenotypically indistinguishable from the single mutants supporting a potential common function. Mutation of the O-fucosylation sites in DLL3 does not disrupt the interaction of DLL3 with LFNG or full length Notch1or DLL1, and O-fucosylation-deficient DLL3 can still inhibit Notch in cis in vitro. However, in contrast to wild type DLL3, O-fucosylation-deficient DLL3 cannot compensate for the loss of endogenous DLL3 during somitogenesis in the embryo. Together our results suggest that the cis-inhibitory activity of DLL3 observed in cultured cells might not fully reflect its assumed essential physiological property, suggest that DLL3 and LFNG act together, and strongly supports that modification of DLL3 by O-linked fucose is essential for its function during somitogenesis.

## Introduction

The Notch signaling pathway mediates local interactions between adjacent cells and thereby regulates developmental processes in a wide variety of different tissues and species [1–6]. Notch receptors and their ligands, so-called DSL-proteins (characterized by a conserved Cysteine-rich region found first in the *Drosophila* Delta, Serrate, and *C. elegans* lag-2 proteins) are transmembrane proteins with multiple EGF-like repeats of varying numbers in their extracellular domains [7–9]. The Notch protein is proteolytically processed and present as a non-covalently linked heterodimeric receptor at the cell surface [10,11]. Upon ligand binding, the intracellular portion of Notch is proteolytically released, translocates to the nucleus, and by complexing with a transcriptional regulator (suppressor of hairless (su(h)) in Drosophila, RBPjk in mouse), activates transcription of a family of bHLH genes [12–18], whose gene products in turn regulate the transcription of downstream effector genes. Activation of Notch through different ligands can be modulated by Fringe proteins, glycosyltransferases that modify Notch in the trans-Golgi [19–21] and can also accept ligands as substrates [22]. Generally, vertebrates contain several copies of genes encoding Notch receptors and ligands. In the mouse, there are three Delta-type (DLL1, DLL3 and DLL4), two Serrate-type (Jagged1 and 2) DSL proteins and four Notch (Notch1-4) receptors. Little is known about how different ligands interact with various Notch receptors, and whether the signals elicited by these interactions are quantitatively or qualitatively different.

In vertebrates, in addition to multiple other processes, somite formation and patterning require Notch signaling [23–27]. Somitogenesis is a patterning process in vertebrate embryos that subdivides the paraxial mesoderm along the anterior-posterior axis into a series of homologous blocks of epithelial cells, the somites. Somites form sequentially on both sides of the neural tube by segmentation of tissues at the anterior end of the unsegmented (the presomitic) paraxial mesoderm (PSM), and are subdivided into cranial and caudal halves, which differ with respect to function [28,29] and gene expression [30–32].

DLL1 and DLL3, two of the mammalian DSL proteins, are coexpressed in the PSM and essential for somitogenesis [33,34]. Like other DSL proteins DLL3 can cis-inhibit Notch when coexpressed with Notch in the same cell [35]. However, in contrast to DLL1 (and the other Notch ligands), DLL3 expressed in cultured cells cannot activate Notch on adjacent cells in vitro [35,36] and, in vivo DLL3 protein expressed instead of DLL1 in mouse embryos did not activate Notch under physiological conditions and failed to compensate for the loss of DLL1 [37]. DLL1 localizes to the cell surface whereas DLL3 resides almost exclusively in the Golgi apparatus both in PSM cells and when overexpressed in cultured cells [36,37], and was suggested to cis-inhibit Notch1 in the PSM by directing full-length Notch1 to late endosome/lysosomes and preventing its S1 processing [36]. Loss of DLL3 function results in a skeletal phenotype which is virtually identical to the phenotype of embryos that lack functional LFNG, a proven modulator of Notch signaling [20,21,38–40]. In the PSM, LFNG negatively regulates Notch [41,42], which may involve both cell autonomous and non-autonomous mechanisms of repression [43]. The apparently identical somite phenotypes of *Dll3* and *Lfng* mutant embryos and their reported inhibitory activity in the PSM raise the possibility that there both gene products together modulate Notch activity.

Here, we report that DLL3 and LFNG interact, DLL3 is O-fucosylated at EGF repeats two and five, and is a substrate for FNG proteins in cultured cells. DLL3 that cannot be O-fucosylated interacts with Notch1, LFNG and DLL1 and retains its cis-inhibitory activity in vitro. However, in contrast to wild type DLL3 O-fucosylation-deficient transgenic DLL3 does not compensate the loss of endogenous DLL3 during somitogenesis in completely ES cell-derived embryos in vivo. Thus, the cis-inhibitory activity of DLL3 on Notch1 that is observed in vitro might not reflect its essential functional property under physiological conditions in the PSM.

## Results

### DLL3 physically interacts with and is modified by FNG in vitro

**Fig 1:**
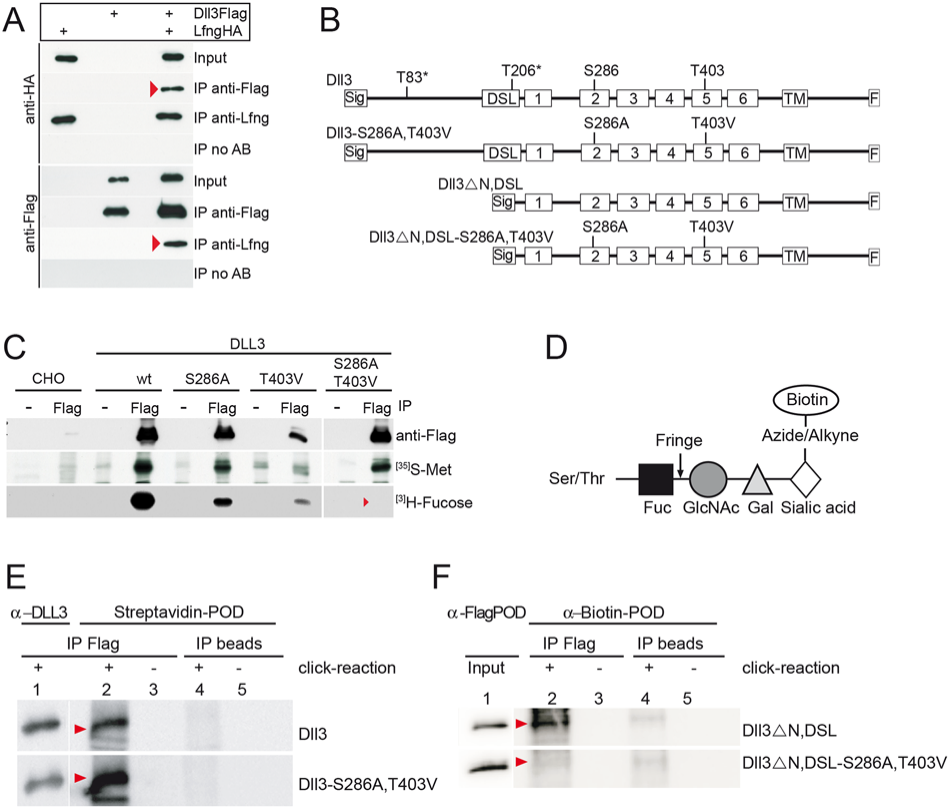
DLL3 physically interacts with LFNG and is modified by POFUT and LFNG at the predicted O-fucosylation consensus motifs in EGF2 and EGF5. (A) Detection of DLL3 and LFNG interaction by coimmunoprecipitations of Flag-tagged DLL3 and HA-tagged LFNG expressed in CHO cells (red arrowheads point to co-precipitated proteins). (B) Schematic overview of expression constructs used for the analysis of DLL3 modifications. DLL3 carries a consensus motif for O-fucosylation (C^2^XXGG(S/T)C^3^) in EGF2 (S at position 286) and EGF5 (T at position 403). T83* and T206* are amino acids in the N-terminal and DSL region predicted to be modified by GalNAc O-glycosylation (NetOGlyc3.1 Server: www.cbs.dtu.dk/services//NetOGlyc, [47]. Sig, Signal sequence; DSL, DSL domain; 1–6, EGF-like repeats 1 to 6; TM, transmembrane region; F, Flag tag, S286A, Serin mutation into Alanin; T403V, Threonin mutation into Valin. (C) Immunoprecipitations of wild type DLL3, DLL3-S286A, DLL3-T403V and DLL3-S286A,T403V proteins from lysates of metabolically labeled CHO cells stably expressing the respective protein. No tritiated fucose was incorporated into the DLL3-S286A,T403V protein (red arrowhead, lower row), indicating that these are the only O-fucosylation sites in DLL3. As a positive control for the metabolic labeling procedure cells were labeled with S^35^-Methionine (middle row). Western blot analysis (upper row) with anti-Flag antibody shows different expression levels of used clones, consistent with different signal intensities after labeling with methionine. (D) Schematic representation of the strategy to analyze O-fucose elongation by LFNG using click-iT chemistry. LFNG catalyses elongation of O-linked fucose (black square) bound to Serin or Threonin in EGF-like repeats with N-Acetylglucosamine (grey circle) followed by Galactose (grey triangle) and Sialic Acid (white diamond) [45]. With the "click" reaction azide modified Sialic acid is chemoselectively ligated to alkyne-tagged Biotin (white circle), which can be detected with Streptavidin or anti-Biotin Antibodies. (E and F) Immunoblots of Flag-tagged DLL3 variants shown in (B) immunoprecipitated from lysates of metabolically Ac_4_ManNAz (sialic acid precursor) labeled CHO cells using anti-Flag antibodies. Incorporation of sialic acid (see D) was visualized with peroxidase-conjugated Streptavidin (E) or with anti-Biotin Antibody (F). Presence of immunoprecipitated DLL3 variant proteins was verified using anti-DLL3 antibodies (E) or on input Lysate with anti-Flag antibodies (F). DLL3 and the O-fucosylation mutant DLL3-S286A, T403V showed incorporation of sialic acid (E, red arrowheads), indicating the presence of additional O-Glycosylation sites. Sialic acid was incorporated into wild type DLL3 lacking the N-terminus and DSL domain (F, upper row, red arrowhead) but not into the truncated variant when S286 and T403 were mutated (DLL3ΔN,DSL-S286A, T403V, see B) indicating further modification of O-fucose residues at S283 and T403.

Previous studies showed that DLL3 and LFNG localize to the Golgi apparatus both in cultured cells and in the PSM. To test whether these proteins physically interact we coexpressed both proteins in CHO cells and analyzed potential physical interactions by immunoprecipitation. Immunoprecipitation of Flag-tagged DLL3 coprecipitated HA-tagged LFNG (upper panel Fig 1A), and immunoprecipitation of HA-tagged LFNG using anti-LFNG antibodies pulled down Flag-tagged DLL3 (lower panel Fig 1A), indicating that both proteins not only reside in the Golgi apparatus but also either directly or indirectly physically interact.

The DLL3 amino acid sequence contains two consensus sites C^2^XXGG(S/T)C^3^ for O-fucosylation by POFUT1 [44,45], S286 in EGF repeat 2 and T403 in EGF5 being the potential acceptor amino acids (Fig 1B). O-linked fucose at EGF repeats in turn can serve as a substrate for fringe proteins, which can add N-acetylglucosamine (GlcNAc) to the fucose residue [19,44]. Colocalization of DLL3 and LFNG in the Golgi apparatus, interaction of DLL3 and LFNG, and the presence of O-fucosylation consensus sites raises the possibility that DLL3 is modified by LFNG, the only fringe protein expressed in the presomitic mesoderm. To test this possibility we first analyzed whether the consensus sites are actually O-fucosylated. Wild type DLL3 and DLL3 variants, in which these sites were mutated individually or in combination, were expressed in CHO cells that express endogenous POFUT1 [46] and DLL3 was immunoprecipitated from lysates of cells that were metabolically labeled with tritiated fucose. Wild type DLL3 as well as the variants containing one mutated O-fucose consensus site incorporated ^[3]^H-fucose (Fig 1C). In contrast, a DLL3 protein carrying mutations at both consensus sites (Dll3-S286A, T403V, Fig 1B) did not incorporate the labeled sugar (Fig 1C), indicating that both sites are O-fucosylated, which is a prerequisite for further modification by fringe proteins.

The elongation of O-linked fucose with GlcNAc cannot be proven directly by metabolic labeling with the fringe substrate UDP-GlcNAc or its precursors, because they are metabolized to substrates of various glycosyltransferases. Therefore, we used an indirect assay to analyze whether DLL3 is a substrate for fringe proteins: After addition of GlcNAc, the O-fuc-GlcNAc disaccharide is further elongated, first by the addition of galactose (Gal), and subsequently by sialic acid (Sia) as was shown in CHO cells (Fig 1D, and [45]). Carbohydrates containing sialic acid can be marked by providing Ac_4_ManNaz (tetraacetylated N-azidoacetyl-D-mannosamine), a modified sialic acid precursor that after conversion to a sialic acid derivative is incorporated instead of sialic acid. After immunopurification of the glycoprotein to be analyzed the sialic acid derivative can be covalently linked to biotin via the, click-chemistry‘ (Fig 1D). Biotin is used for subsequent detection in Western blots. To specifically detect elongation of fringe modified proteins by this strategy requires that no other carbohydrate side chains that contain sialic acid are present. Analysis of the DLL3 amino acid sequence using the NetOGlyc 3.1 Server (www.cbs.dtu.dk/services/NetOGlyc, [47] predicted two mucin type GalNAc O-glycosylation sites in the N-terminal and DSL region of DLL3 (T83* and T206* in Fig 1B). To test whether these and/or other sites may be used to link sialic acid-containing carbohydrates, we metabolically labeled CHO cells stably expressing LFNG and transiently expressing tagged wild type DLL3 and DLL3-S286A,T403V with Ac_4_ManNaz and analyzed the DLL3 proteins after immunoprecipitation and biotinylation. Both proteins were linked to biotin (Fig 1E, lane 2), indicating that sialic acid is incorporated into carbohydrates that are not O-fucose linked to EGF 2 and 5. Therefore, we generated a DLL3 protein from which the N-terminal region including the DSL domain was deleted (Dll3ΔN,DSL, Fig 1B). This DLL3 variant was still metabolically labeled (Fig 1F, lane 2, upper row). In contrast, the N-terminally truncated DLL3 protein, in which the O-fucosylation consensus sites were mutated (Dll3ΔN, DSL-S286A,T403V; Fig 1B), no longer incorporated Ac_4_ManNaz-derived sialic acid (Fig 1F, lane 2 lower row), indicating that the O-fucose-linked residues in EGF 2 and/or 5 are elongated by fringe proteins and subsequent sugar additions. We conclude that DLL3 is a substrate for and modified by fringe in cultured cells in vitro. Since CHO cells express endogenous LFNG as well as RFNG [48] both enzymes could contribute to elongation of O-linked fucose in our assay.

### *Dll3* and *Lfng* null mutations do not act synergistically

Both DLL3 and LFNG were reported to act as negative regulators of Notch signaling in the PSM [35,36,41,43]. However, it is unclear whether both proteins act independently or one requires the function of the other for this regulation. If DLL3 and LFNG function would depend on each other the simultaneous removal of both proteins should not affect the severity of the somite patterning defects observed in the single mutants. Conversely, if the activities of DLL3 and LFNG in the presomitic mesoderm affect Notch activity independently one could expect an additive effect leading to an enhancement of the individual mutant phenotypes in double mutant embryos.

**Fig 2:**
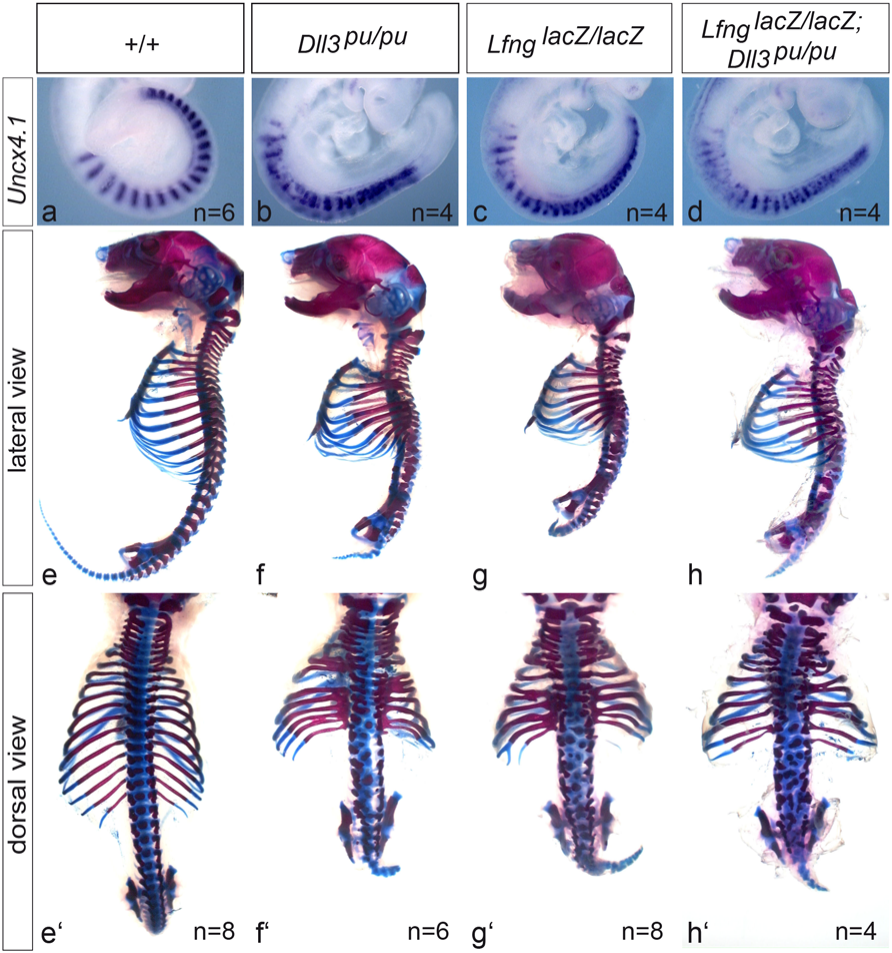
Somite patterning and vertebral column defects in single and double *Dll3* and *Lfng* mutants. Whole-mount in situ hybridizations of E9.5 embryos with an *Uncx4.1* specific probe (a-d) and lateral (e-h) and dorsal (e‘-h‘) views of skeletal preparations of wild type (a, e,e‘), homozygous *Dll3* ^*pu*^ (b, f, f‘), homozygous *Lfng* ^*lacZ*^ (c, g, g‘) and double homozygous *Dll3* ^*pu*^; *Lfng* ^*lacZ*^ E18.5 embryos (d, h, h‘). Absence of both proteins does not enhance somite A-P patterning or vertebral column defects present in single mutants.

To obtain genetic support for either of these possibilities we generated double homozygous mutants and analyzed somite patterning in E9.5 embryos using *Uncx4.1* expression as a marker for anterior-posterior (A-P) patterning, and axial skeleton preparations at E18.5. In wild type *Uncx4.1* is expressed exclusively in caudal somite compartments leading to a highly regular striped pattern (Fig 2a). *Dll3/Lfng* double heterozygous embryos were indistinguishable from wild type (data not shown). Both, in *Dll3* and *Lfng* null mutants the expression pattern of *Uncx4.1* is irregular and variable between embryos, and *Uncx4.1* expressing cells are not restricted to caudal compartments (Fig 2b and 2c). In contrast to Zhang et al [49] who reported a less severe disruption of the normal *Uncx4.1* expression pattern in *Dll3* mutant than in *Lfng* mutant embryos, we observe a similar range of abnormal *Unc4.1* expression in the single mutants (Fig 2b, 2c and S1 Fig). In *Dll3/Lfng* double homozygous mutant embryos the *Uncx4.1* expression pattern was similarly disrupted, varied between embryos and was indistinguishable from the single mutants (Fig 2d and S1 Fig). The axial skeletons of both *Dll3* and *Lfng* homozygous mutants are shortened, with numerous rib and vertebral fusions, and highly disorganized vertebrae (Fig 2f, 2f‘ and 2g, 2g‘ and [33,38,40,50]). Very similar defects were observed in skeletal preparations of *Dll3/Lfng* double homozygous mutants (Fig 2h and 2h‘). Collectively, *Uncx4.1* expression and axial skeleton preparations indicate virtually identical phenotypes in *Dll3* and *Lfng* single and double mutants. Thus, loss of both proteins does not cause recognizable additive or synergistic effects as can be observed in other mutants affecting Notch signaling (e. g. [37,51]) supporting the idea that both proteins might act functionally together.

### DLL3 that cannot be O-fucosylated interacts with NOTCH1 and LFNG, and *cis*-inhibits Notch1 in vitro

**Fig 3:**
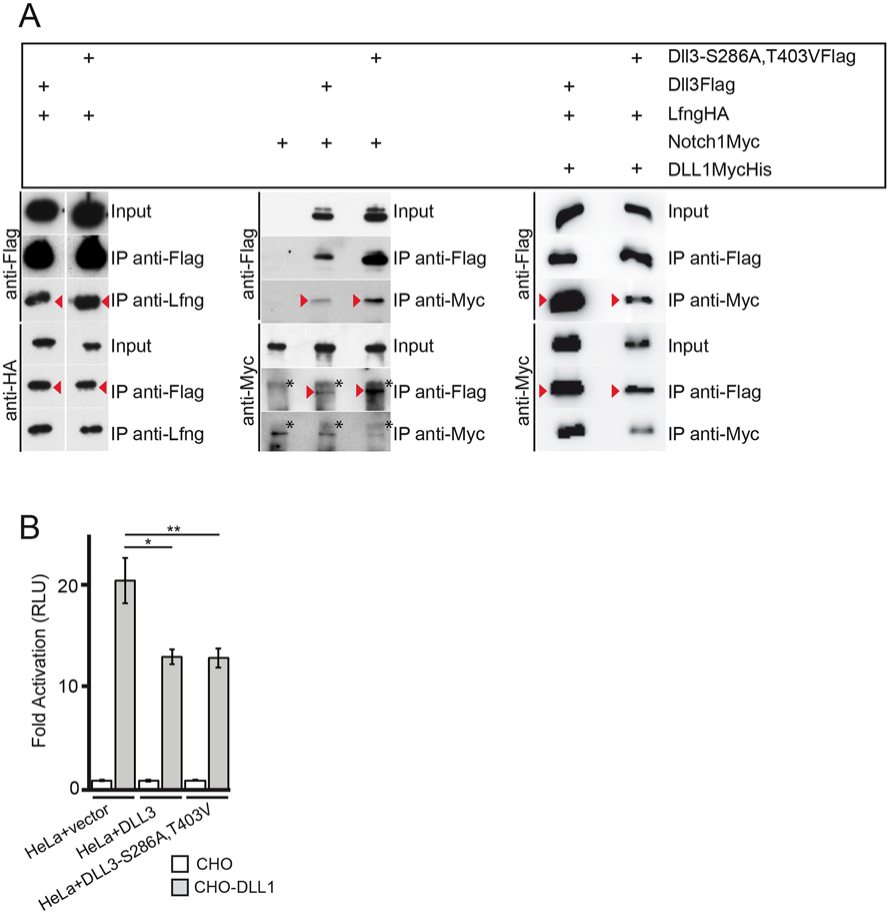
O-fucosylation is not essential for DLL3 interaction with LFNG, full length Notch1 and DLL1, and Notch1 *cis*-inhibition. (A) Flag-tagged wild type DLL3 or DLL3-S286A, T403V, and HA-tagged LFNG, Myc-tagged Notch1 and MycHis-tagged DLL1, respectively, were immunoprecipitated from lysates of CHO cells expressing the indicated combinations of these proteins demonstrating that the O-fucosylation defective DLL3 S286A, T403V protein interacts with LFNG, full length Notch1 and DLL1 (red arrowheads). The protein running above full length Notch (marked by asterisks) represents a background band recognized by the anti-Myc antibody in immunoprecipitated material. (B) Notch transactivation assay. HeLa cells, transfected with the (4xRBP-Jκ)-luciferase reporter, Renilla luciferase and expression vectors for DLL3, DLL3-S286A,T403V or empty vector respectively, were cocultivated either with wild type CHO cells or CHO cells stably expressing DLL1. Luciferase activity from HeLa cocultured with CHO cells (set to 1) and CHO cells stably expressing DLL1 were measured and normalized to the expression levels of the appropriate constructs. Error bars represent SD. * = p<0.05; **0 p<0.01.

DLL3 interacts with full length Notch1 in cis [35,36] and this interaction was suggested to inhibit Notch in vitro and in vivo [36]. To analyze whether this interaction depends on O-fucosylation an expression vector for Myc-tagged Notch1 was cotransfected with Flag-tagged DLL3 wt or Flag-tagged DLL3-S286A,T403V expression plasmids, respectively, and proteins were immunoprecipitated using anti-Myc or anti-Flag antibodies. Both, wild type DLL3 and mutant DLL3-S286A,T403V protein coprecipitated full length (uncleaved) Notch1 and vice versa (Fig 3A, arrowheads middle panel), indicating that the amino acid exchanges in DLL3 did not perturb this interaction and O-fucosylation of DLL3 is not essential for binding to Notch1, which is consistent with the observation that DLL3-Notch1 protein complexes can be formed regardless of Lfng is present or not [35]. Likewise, O-fucosylation-deficient DLL3 still interacted with LFNG (Fig 3A, arrowheads left panel), indicating that recognition of DLL3 by LFNG also does not depend on the presence of O-linked fucose. Next, we tested whether O-fucosylation of DLL3 is required for its ability to inhibit NOTCH1 *in cis* in HeLa cells expressing endogenous NOTCH1 [52]. HeLa cells transfected with an RBP-Jκ-luciferase reporter and cocultured with DLL1 expressing CHO cells activated the RBP-Jκ-luciferase reporter about 20 fold compared to cocultivations with wild type CHO cells (Fig 3B). Expression of wild type DLL3 in HeLa cells consistently reduced the activation of the reporter to approximately 13 fold, which corresponds to a reduction of about 40% (Fig 3B). A virtually identical reduction was observed with the DLL3-S286A,T403V variant in two independent experiments, suggesting that O-fucosylation at EGF repeats 2 and 5 is not essential for the *cis*-inhibitory properties of DLL3 under these assay conditions. Thus, the amino acid changes in DLL3 that prevent O-fucosylation do not affect major biochemical characteristics concerning protein interactions and the cis-inhibitory activity of DLL3 in vitro.

### DLL3 interacts with DLL1 and affects its subcellular localization in vitro

**Fig 4:**
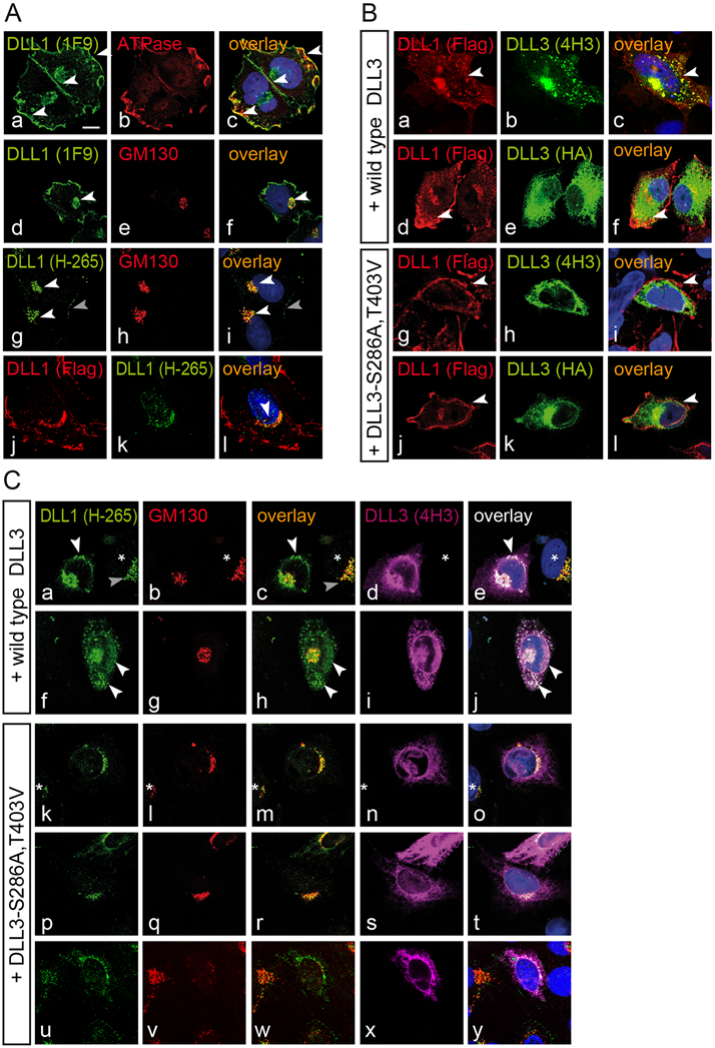
Wild type DLL3 but not DLL3-S286A,T403V affects the subcellular localization of DLL1. Confocal images of CHO cells stably expressing Flag-tagged DLL1 (A), transfected either with HA-tagged wild type or fucosylation mutant DLL3 (B, C). (A) Costaining of DLL1-Flag (using MAb 1F9) with the cell surface marker Sodium potassium ATPase (a-c) detects DLL1 on the cell surface (arrowheads in a and c), costaining with GM130 (d-f) in the trans-Golgi (arrow heads in d and f). In contrast, staining of DLL1-Flag using anti-DLL1 antibody H-265 detects predominantly Golgi localized DLL1 (white arrowheads in g and i) and reacts only weakly with DLL1 on the cell surface (g and i, grey arrowhead). DLL1 staining with anti-Flag detects DLL1-Flag similar to staining with 1F9 (compare j with a or d) overlapping with H-265 staining in the Golgi (arrowhead in l). (B) Coexpression of wild type DLL3 in DLL1 CHO cells (a-f) leads to an intracellular accumulation of DLL1 protein (white arrowheads in a, c and d, f), whereas in cells coexpressing the fucosylation mutant of DLL3 (g-l) localization of DLL1 is similar to untransfected cells and readily detected at the cell surface (white arrowheads in g, i and j, l). (C) Coexpression of wild type DLL3 with DLL1 protein leads to colocalization of DLL3 and DLL1 in vesicular structures or punctae outside the Golgi (white arrowheads in a, c, e, f, h, j; 18 out of 21 cells). The asterisk in a-e marks a neighboring DLL1 expressing cell without DLL3 expression showing intracellular DLL1 protein confined to the trans-Golgi (grey arrowhead in a and c). Coexpression of the O-fucosylation-defective DLL3 mutant (k-t) had no obvious effect on the localization of intracellular DLL1 protein (k, m, o, p, r, t; 10 our of 14 cells) or resulted only in weak detection of DLL overlapping with DLL3 outside the Golgi (u-y). The asterisk in k-o marks a neighboring DLL3 non-expressing cell.

Recently, LFNG has been shown to repress DLL1 function in NIH3T3 cells that simultaneously express DLL3 and Notch1 („trans-repression”[43]), whereas coexpression of DLL3 with DLL1 in L cells had no effect on DLL1-mediated Notch activation [35]. To get potential hints as to how DLL3, which is coexpressed with DLL1 in the Golgi apparatus [37], might affect DLL1, and whether O-fucosylation might play a role we analyzed the localization of stably expressed Flag-tagged DLL1 in CHO cells (which express endogenous LFNG [53]) in the absence or presence of transiently expressed wild type DLL3 or DLL3-S286A,T403V. In CHO cells stably expressing Flag-tagged DLL1 the protein was detected on the cell surface (arrowheads in Fig 4Aa) overlapping with Sodium/Potassium ATPase (arrowheads in Fig 4Ac), a marker for the plasma membrane [54], as well as intracellularly (white arrowheads in Fig 4Ad and 4Ag) overlapping with the Golgi marker GM130 (white arrowheads in Fig 4Af and 4Ai). In cells overexpressing DLL3 the clear membrane staining was less obvious and there was an apparent intracellular accumulation of DLL1 staining (arrowheads in Fig 4Ba, 4Bc, 4Bd and 4Bf). In cells overexpressing the DLL3-S286A,T403V protein DLL1 clearly localized at the membrane (arrowheads in g, i, j, l) and no obvious intracellular accumulation of DLL1 was detected. Since the monoclonal anti-DLL1 (from rat) and anti-Flag (from mouse) antibodies used in these initial experiments cannot be used to simultaneously stain with anti-DLL3 (from rat) and anti-GM130 (from mouse), we performed additional staining with the polyclonal anti-DLL1 antibody H-265 (from rabbit), which is directed against an epitope in the C-terminal region. This antibody preferentially detected Golgi-localized Flag-tagged DLL1 (white arrowheads Fig 4Ag and 4Ai) overlapping with anti-Flag staining (arrowhead in Fig 4Al) and reacted only weakly with DLL1 on the cell membrane (gray arrowhead in Fig 4Ag and 4Ai). Also with the anti-DLL1 H-265 antibody we detected an increase of intracellular DLL1 staining in cells overexpressing wild type DLL3 (arrowheads in Fig 4Ca, 4Cc, 4Ce, 4Cf, 4Ch and 4Cj). Intracellular DLL1 staining partly overlapped with GM130 and to a large extent in a punctate or vesicular pattern with overexpressed DLL3 outside the Golgi apparatus (18 out of 21 cells; arrowheads in Fig 4Ce and 4Cj; note that the neighboring cell in (e) not expressing DLL3 (marked by an asterisk) shows no DLL1 staining outside the Golgi apparatus). In contrast, in cells overexpressing the DLL3-S286A,T403V variant the intracellular DLL1 staining did not change obviously (10 out of 14 cells; Fig 4Ck–4Ct) or only weak DLL1 staining was detected in regions where mutant DLL3 accumulated (Fig 4Cu–4Cy). The apparent relocalization of DLL1 by wt but not mutant DLL3 might be explained by differential interaction of wt and mutant DLL3 with DLL1. To test this possibility we performed co-immunoprecipitation experiments with stably expressed Flag-tagged DLL3 or mutant DLL3, and transiently expressed MycHis-tagged DLL1 in CHO cells (Fig 3A). Both wt DLL3 and mutant DLL3 coprecipitated DLL1 (arrowheads in Fig 3A, right panel). To test whether wt and mutant DLL3 differ in their ability to inhibit DLL1 we cocultured CHO cells stably expressing DLL1 and either wt or mutant DLL3 transfected with or without an LFNG expression vector with HeLaN1 cells transiently expressing LFNG and measured the activation of a Notch reporter. In two independent experiments both wt and mutant DLL3 had only marginal effects on Notch activity independent of the presence of exogenous LFNG (data not shown). Thus, overexpression of wt but not mutant DLL3 can affect the subcellular localization of DLL1, and both DLL3 proteins interact with DLL1 but have no significant effect on DLL1 activity under our assay conditions.

### O-fucosylation-defective DLL3 does not function in vivo

**Fig 5:**
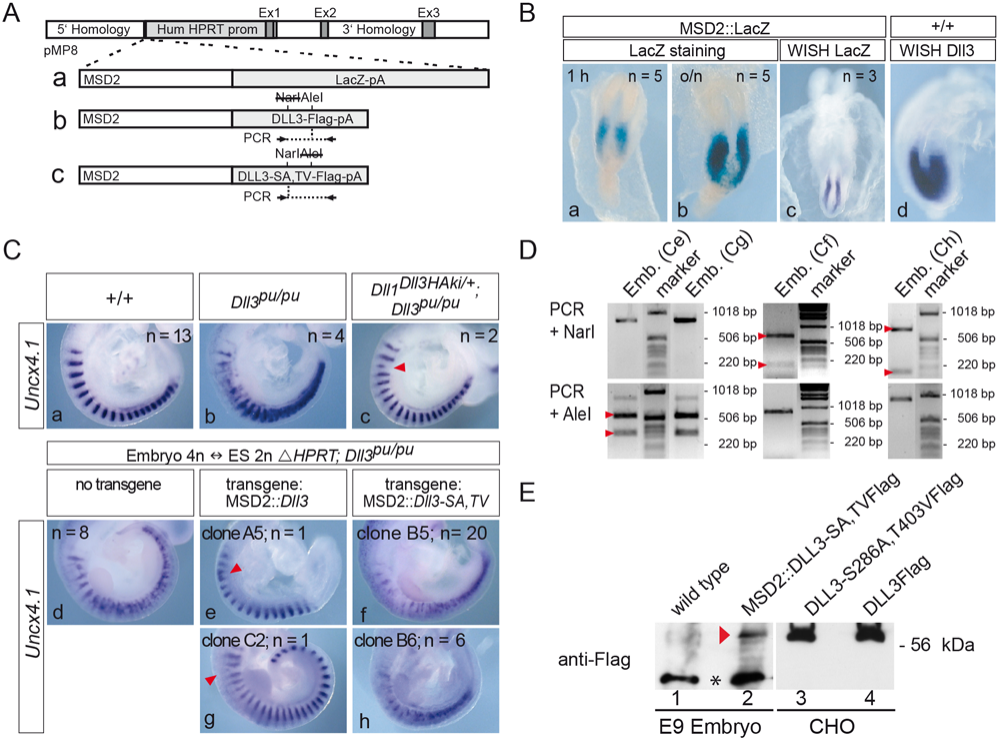
The DLL3-S286A,T403V mutant does not complement the loss of endogenous DLL3 in somitogenesis. (A) Schematic representation of constructs introduced into HPRT deficient homozygous *Dll3* mutant ES cells. MSD2 is a dimer of the mesoderm-specific promoter element (MSD) from the *Dll1* locus [59]. Locations of primers and restriction sites used for genotyping of embryos derived from tetraploid complementation are indicated below and above schemes b and c, respectively. (B) The MSD2 promoter element drives transgene expression in the PSM similar to endogenous *Dll3* expression. E8.5 MSD2::LacZ chimeric embryos were stained for β-galactosidase activity for one hour (a) and over night (b), or were analyzed by whole-mount in situ hybridization with a *lacZ* specific probe (c). (d) Expression pattern of endogenous *Dll3* transcripts in an E8.5 wt embryo for comparison. (C) Whole mount in situ hybridizations of E9.5 wt (a), homozygous *Dll3* ^*pu*^ (b), and *Dll1* ^*Dll3ki*^, *Dll3* ^*pu/pu*^ [38] (c) embryos. In *Dll1* ^*Dll3ki*^, *Dll3* ^*pu/pu*^ embryos, which lack endogenous DLL3 but express DLL3 from the *Dll1* locus, expression of the anterior-posterior (A-P) somite patterning marker *Uncx4.1* is restored except for minor irregularities (arrowhead in c). (d-h) Completely ES cell-derived embryos hybridized with *Uncx4.1*. Embryos generated from ES cells homozygous mutant for *Dll3* (*Dll3* ^*pu/pu*^) and carrying the HPRT deletion (ΔHPRT) display the same A-P somite patterning defects as homozygous *Dll3* ^*pu*^ embryos (compare d with b). Expression of wild type DLL3 in ES cell-derived embryos almost completely rescues the *Dll3* ^*pu*^ A-P somite patterning defect except for minor irregularities (arrowheads in e, g), whereas ES cell-derived embryos expressing mutant DLL3 display a *pudgy*-like somite phenotype (f, h) indicating that DLL3-S286A, T403V is not functional during somitogenesis. (D) Genotyping of tetraploid embryos shown in (C) using PCR and restriction digests as indicated in (A). The wild type *Dll3* PCR product is cut by *AleI* but not *NarI* (left panel), whereas the *Dll3*-S286A, T403V PCR product is cut by *NarI* (due to the presence of S286A) but not by *AleI* (due to the presence of T403V; middle and right panel; see Material and Methods for further details). Letters in parentheses refer to embryos shown in (C), arrowheads indicate cleavage products of the expected sizes. (E) Western blot analysis of lysates of wild type embryos (lane 1) or embryos obtained with ES cell clone B5 (lane 2) and lysates of CHO cells overexpressing Flag-tagged DLL3-S286A, T403V (lane 3) or wild type DLL3 (lane 4) using anti-Flag antibodies confirmed expression of DLL3-S286A, T403V-Flag in completely ES cell-derived embryos (red arrowhead). The equivalent of 4 trunks of E9 embryos was loaded in lanes 1 and 2. Asterisk between lanes 1 and 2 indicates a background band detected in embryo lysates.

The in vitro assays might not allow to distinguish the activity of wt and O-fucosylation-deficient DLL3 in vivo. To test whether O-fucosylation is important for DLL3 under physiological conditions during somitogenesis we devised a strategy to test for DLL3 function in vivo. This strategy is based on our previous finding that expression of *Dll3* targeted to the *Dll1* locus (*Dll1* ^*Dll3ki*^) is able to rescue loss of endogenous DLL3 [37], and on the possibility to generate completely ES cell-derived embryos by tetraploid complementation [55]. We established ES cells that are homozygous for the *Dll3* ^*pu*^ null allele [33] and carry the E14tg2a deletion at the HPRT locus (*ΔHPRT*; *Dll3* ^*pu/pu*^) and are thus HAT sensitive [56]. These cells allow for efficient integration of single copy transgenes into the HPRT locus by homologous recombination using the targeting vector pMP8 (Fig 5A). Correct targeting restores HPRT activity and renders E14TG2a cells HAT resistant, and allows for faithful expression of transgenes from heterologous promoters [57,58].

First, we confirmed that a dimerized MSD element (MSD2) of the *Dll1* promoter [59] when inserted into the HPRT locus specifically and efficiently directs *lacZ* expression into the PSM (for a schematic representation of construct see Fig 5Aa), in a pattern virtually identical to expression of *Dll3* (Fig 5B; note that the different staining intensities observed in the WISHs are from different experiments using different probes and do not allow to compare expression levels). Next we established that *ΔHPRT*; *Dll3* ^*pu/pu*^ ES cells give rise to completely ES cell-derived embryos after injection into tetraploid wild type embryos, and ascertained the *Dll3* ^*pu/pu*^ phenotype by WISH using an *Uncx4.1* probe (compare Fig 5Cb and S1a–S1c Fig with Fig 5d and S3a–S3c Fig). Subsequently, we tested whether a construct directing expression of wild type DLL3 under the control of the MSD2 element (Fig 5Ab) in *Dll3* ^*pu/pu*^ E14tg2a ES cells (ΔHPRT; MSD2::Dll3; *Dll3* ^*pu/pu*^) can rescue the pudgy phenotype in ES cell-derived embryos. Embryos derived from two independently generated ΔHPRT; MSD2::Dll3; *Dll3* ^*pu/pu*^ ES cell lines (clone A5 and C2) had only minor somite patterning abnormalities (arrowheads in Fig 5Ce and 5Cg) similar to what was observed in heterozygous *Dll1* ^*Dll3ki*^ embryos lacking *Dll3* function (arrowhead in Fig 5Cc). This indicated that expression levels obtained with the MSD2 element in the HPRT locus are sufficient to rescue the lack of endogenous DLL3. Having established that this strategy can successfully be used to analyze the functionality of DLL3 variants during somitogenesis in vivo we applied this approach to DLL3-S286A,T403V, and analyzed tetraploid embryos derived from two independent ES cell clones carrying the MSD2::DLL3-S286A,T403V expression construct (Fig 5Ac). In contrast to wild type DLL3, DLL3-S286A,T403V expressed under the MSD2 element did not restore normal *Uncx4.1* expression (Fig 5Cf and 5Ch). Similar to *Dll3* ^*pu/pu*^ embryos we observed variable abnormal *Uncx4.1* expression patterns in MSD2::DLL3-S286A,T403V tetraploid embryos (S3 Fig), indicating that DLL3-S286A,T403V did not rescue the loss of endogenous DLL3 and behaved like the null allele. The genotype of tetraploid embryos was ascertained by allele-specific PCRs (Fig 5D and data not shown), and expression of the DLL3-S286A,T403V protein in tetraploid chimeras was verified by Western blot analysis using anti-Flag antibodies (Fig 5E). To test whether significantly different protein stability might contribute to the inability of DLL3-S286A,T403V to rescue the loss of endogenous DLL3 we compared the stability of wt DLL3 and mutant DLL3 in cycloheximid-treated CHO cells stably expressing wt or mutant DLL3 as well as LFNG. LFNG levels dropped during this period whereas both wt and mutant DLL3 were similarly stable and declined only marginally over a 5 hr period (S2 Fig), indicating that the introduction of the S286A and T403V mutations into DLL3 does not obviously destabilize the DLL3 protein in CHO cells. Thus, a DLL3 variant that still interacts with and cis-inhibits NOTCH1in vitro is not functional in vivo, suggesting that O-fucosylation of DLL3 by Pofut1, and potentially also subsequent modification by LFNG is essential for DLL3 function in the PSM in vivo.

## Discussion

In this study we have established that DLL3 is O-fucosylated, is a substrate for fringe proteins and interacts with LFNG in vitro. O-fucosylation-deficient DLL3 still interacts with Notch1 and retains major biochemical properties as well as its cis-inhibitory activity in vitro. However, a DLL3 variant that cannot be O-fucosylated is not functional in vivo. Thus, our results uncover different activities of DLL3 in vitro and in vivo and suggest that *cis*-inhibition of Notch is not the sole function of DLL3 in vivo.

The DLL3 variant that cannot be O-fucosylated did not complement the loss of DLL3 in the PSM, suggesting that O-fucosylation is essential for DLL3 function in vivo. Formally we cannot exclude that the exchange of the respective serine or threonine residue accepting *O*-fucose rather than the actual loss of *O*-fucosylation could affect DLL3 function due to misfolding of the mutant protein. However, the mutant protein retained major biochemical and biological properties in vitro, i. e. binding to full length Notch1, LFNG and DLL1, and cis-inhibition. In our in vivo experiments MSD2::DLL3 expression was sufficiently high to complement the loss of endogenous DLL3, although we do not know how the levels of endogenous and transgenic DLL3 compare. Mutant DLL3 protein was present in completely ES-cell-derived embryos, and the stability of DLL3 and DLL3-S286A,T403V was similar in cultured cells in vitro, indicating that the inability of the MSD2::DLL3-S286A,T403V construct to rescue the pudgy phenotype cannot be attributed to absence of the mutant protein or obviously altered protein stability. Thus, we favor the explanation that O-fucosylation (and potentially further modification) is important for DLL3‘s physiological function. which implies that DLL3 is also a substrate for POFUT1 in vivo. There it is likely modified at both consensus sites, since it has been observed that all consensus sites in EGF repeats of Notch1 are modified by POFUT1 [44,60]. A role for O-fucosylation might also be supported by the somite phenotype of a hypomorphic *Pofut1* allele, which specifically affects somitogenesis [61], although currently we cannot distinguish between effects on Notch receptors and DLL3 in this *Pofut1* mutant. In *Drosophila* O-fucosylation was dispensable for ligand function in the imaginal wing disc [62], and mouse DLL1, which is O-fucosylated at four consensus sites, was functional in POFUT1-deficient cells [63]. Thus, DLL3 appears to be the first DSL protein whose physiological function requires O-fucosylation.

Not all O-fucosylated EGF repeats are substrates for fringe proteins as has been shown for O-fucosylated EGF repeats in Notch1 [44]. However, at least one of the O-fucosylation sites of DLL3 is a substrate for further modification by fringe proteins in vitro. Therefore, DLL3 is also a likely substrate under physiological conditions in vivo. Of the three mammalian fringe proteins only LFNG is expressed in the PSM [64] suggesting that in vivo DLL3 is a substrate for LFNG, which raises the possibility that LFNG exerts (part of) its function through modification of DLL3, which is supported by the highly similar phenotypes of null alleles of both genes and the interaction of overexpressed DLL3 and LFNG in vitro, which raises the possibility that DLL3 and LFNG also interact in the PSM. There are no antibodies available that allow one to detect endogenous LFNG in mouse embryos, and even an HA-tagged LFNG protein overexpressed in the PSM [65] could not be detected (data not shown), which currently precludes to demonstrate the interaction between DLL3 and LFNG in vivo. Whether the interaction observed in cultured cells in vitro is direct or mediated by (an)other as yet unidentified protein(s) remains to be analyzed. O-fucosylation was not essential for interaction of DLL3 with Notch1, LFNG and DLL1 in vitro, but was essential for DLL3‘s in vivo function, implying that the mere interaction of DLL3 with Notch1/LFNG/DLL1 is not sufficient for the physiological function of DLL3. The interaction of non-fucosylated DLL3 with Notch1 implies that also further modification by LFNG is not critical for interaction of DLL3 with Notch1, albeit we cannot exclude that under physiological conditions in vivo there might be differences in binding strength or interaction sites depending on the presence of O-fucose and additional sugar moieties.

O-fucosylation-deficient DLL3 that was non-functional in vivo retained its cis-inhibitory activity in vitro. This suggests that cis-inhibition of Notch by DLL3 observed in vitro is not sufficient for and does not fully reflect the essential physiological function of DLL3 in the PSM. However, relative expression levels of Notch and DLL3 might differ between HeLa and PSM cells. Thus, we cannot exclude that DLL3-S286A,T403V is expressed in HeLa cells at levels that still can cis-inhibit, whereas in the PSM these levels are below a certain threshold and under these conditions O-linked fucose might be required for cis-inhibition.

The recently reported observation that DLL3 together with LFNG can inhibit DLL1 from activating NOTCH1 in trans, whereas coexpression of LFNG with DLL1 had only a mild effect on DLL1 activity [43] and coexpression of DLL3 alone with DLL1 did not attenuate Notch activation in neighboring cells [35,43], appeared as an appealing explanation to reconcile our in vitro and in vivo data. Consistent with the idea that DLL3 can modulate DLL1 activity we observed intracellular retention of DLL1 when wt DLL3 was coexpressed in the presence of LFNG, and found co-immuno precipitation of DLL1 and DLL3. Co-expression of DLL3-S286A,T403V did not relocalize DLL1, suggesting that sugar modification of DLL3 is important. However, also DLL3-S286A,T403V could be co-immuno precipitated with DLL1, and we did not observe any significant effect of either wt or mutant DLL3 in the presence or absence of LFNG on the ability of DLL1 to activate Notch under our assay conditions. Thus, the (direct or indirect) interaction of DLL3 with DLL1 cannot explain the distinct effects of wt DLL3 and DLL3-S286A,T403V on the subcellular localization of DLL1, and the importance of O-linked fucose on DLL3 for trans-repression remains to be analyzed further.

Null alleles of *Dll3* or *Lfng* have virtually identical ranges of phenotypes concerning somite compartmentalization and axial skeleton, suggesting a common function(s). However, there are also reported differences concerning the effects of these mutations. In *Lfng* mutants elevated graded static Notch activation [41,43] as well as dynamic Notch activation in the PSM were reported based on analyses of sections [66]. Our whole mount analysis of *Dll3* mutant embryos [37] showed static but not obviously enhanced Notch activation, which might be explained by different sensitivities. In addition, expression of the Notch target *Nrarp* appeared to be less affected in the PSM of *Lfng* mutant embryos than in *Dll3* mutants [67]. Thus, despite the indistinguishable phenotypic outcome concerning somite compartmentalization DLL3 and LFNG might also have specific functions that affect Notch activation differently in the PSM.

In conclusion, we have shown that DLL3 directly or indirectly interacts with LFNG, is O-fucosylated, and a substrate for further modification by fringe proteins in vitro, which suggests that in the PSM DLL3 is modified by LFNG. Modification by O-linked fucose is not essential for interaction of DLL3 with Notch1, LFNG or DLL1 and for cis-inhibition in vitro, but appears to be critical for DLL3 function in vivo. This suggests that cis-inhibition of NOTCH by DLL3 does not fully reflect its physiological role and warrants further analyses of the biochemical function of this intracellular DSL protein.

## Materials and Methods

### Ethics Statement

All animal experiments were performed according to the German rules and regulations (Tierschutzgesetz) and approved by the ethics committee of Lower Saxony for care and use of laboratory animals LAVES (Niedersächsisches Landesamt für Verbraucherschutz und Lebensmittelsicherheit). Mice were housed in the central animal facility of Hannover Medical School (ZTL) and were maintained as approved by the responsible Veterinary Officer of the City of Hannover. Animal welfare was supervised and approved by the Institutional Animal Welfare Officer (Tierschutzbeauftragter).

### Skeletal preparations of E18.5 embryos

E18,5 embryos were prepared, stained and documented as described previously [37].

### Genotyping of mutant embryos

Genomic DNA isolated from yolk sacs of E9.5 or liver of E18,5 embryos was used for PCR-genotyping. Detection of the *pudgy* allele [33] was done with PCR primer pair dll3pu1 ACGAGCGTCCCGGTCTATAC and dll3pu2 AGGTGGAGGTTGGACTCACC, which amplifies a 100 bp PCR-product. Restriction digest with *Hae*III generates a 65 bp fragment that is specific for the wild-type allele. *Lfng* mutant mice [40] were genotyped with an allele-specific PCR with the following primer combinations: lfhs1 GAACAAATATGGCCATTCACTCCA and lfgwF13 GGTCGCTTCTCGCCAGGGCGA amplifies a 500 bp PCR-product from the wild-type allele, whereas PCR primer pair lfwF2 CCAAGGCTAGCAGCCAATTAG and lacZB2 GTGCTGCAAGGCGATTAAGTT generates a 450 bp fragment from the mutant allele.

### Whole-mount in situ hybridization

Whole-mount in situ hybridization was performed with digoxygenin-labeled antisense riboprobes as described [68]. Documentation was done with the Leica M420 microscope with Apozoom 1:6 and the Photograb-300Z version 2.0 software. Probes used were *Uncx4.1* [30] and *Dll3* [69]. A *lacZ*-specific riboprobe was generated from a 3 kb ClaI/XbaI Fragment from the *LacZ* gene cloned into pBluescript II KS (Stratagene) using T7 RNA Polymerase.

### Generation of expression constructs and stably expressing cell lines

The DLL3Flag-pTracer-IRESneo expression construct was described previously [37]. To obtain mutated DLL3 variants cDNA fragments containing mutant fucosylation sites were generated by gene synthesis and introduced into the wild-type *Dll3* expression construct by conventional cloning. To obtain the S286A mutation the AGC triplet was changed to GCC resulting in an additional NarI restriction site (see Fig 5A). For the T403V mutation ACG was changed into a GTG triplet, which eliminates an AleI site (see Fig 5A). HA-tagged mouse Lunatic fringe [65], was inserted as a EcoRI/SpeI fragment into the pTracer-CMV expression vector. The Notch1Myc-pCS2+ expression construct containing AA 1 to 2184 of mouse Notch1 followed by a 6-fold Myc-tag was kindly provided by Raphael Kopan. CHO cell lines stably expressing HA-tagged Lfng and Flag-tagged DLL3 or DLL3 fucosylation mutant versions were generated as described previously [37].

### Coimmunoprecipitation

For coimmunoprecipitation cells from a confluent 10 cm culture dish were washed once with cold PBS and harvested in 500 μl lysisbuffer (50 mM Tris-HCl, pH 7,5; 150 mM NaCl; 1 mM EDTA, pH 8,0; 1% NP-40; 1% TritonX-100 with Protease Inhibitor cocktail tablet (04 693 159, Roche)) on ice. After homogenization with a 25-gauge needle protein lysates were cleared by centrifugation at 12,000 g for 10 min at 4°C. For the input control a 50 μl aliquot was taken and mixed with 2 x sample buffer. The remaining lysate was used for immunoprecipitation with the appropriate antibody and Protein G Ultralink Resin (Thermo Scientific) over night at 4°C. Ultralink beads were washed three times with IP washing buffer (50 mM Tris-HCl pH8,5; 500 mM NaCl; 5 mM EDTA pH 8,0, 0,05% NP-40, 1 mg/ml BSA and Protease Inhibitor cocktail tablet) and finally resuspended in 2 x sample buffer (125 mM Tris-HCl pH 6.8, 20% Glycerol, 4% SDS, 5% β-Mercaptoethanol, 0,025% Bromphenolblue) for SDS-PAGE and Western blot analysis.

### Antibodies

Antibodies used for Western blot analysis and coimmunoprecipitation were anti-HA-POD (clone 3F10, 12158167, Roche), anti-Flag-POD (M2, A8592, Sigma), anti-Flag (M2, F3165, Sigma), anti-Myc (clone 9E10, M5546, Sigma) and anti-Lfng (A-19, sc-8239, Santa Cruz Biotechnology, Inc.), and a rat monoclonal anti DLL3 antibody 4H3 (generated against a peptide comprising amino acids 557-571of mouse DLL3) as described in [37]. Primary antibodies used for immuncytochemistry were anti-Flag (M2), anti-HA (3F10), anti-GM130 (610823,BD Biosciences), anti-Alpha 1 sodium potassium ATPase (ab7671, Abcam), anti-DLL3 (4H3), anti-DLL1 (H-265, sc-9102, Santa CruZ Biotechnology, Inc.) and anti-DLL1 (1F9, [37]. Secondary antibodies used were: goat anti-mouse-Alexa633 (A21052, Invitrogen), goat anti-rat-Alexa488 (A11006, Invitrogen), goat anti-rabbit-Alexa488 (A11034, Invitrogen), goat anti-rat-Alexa555 (A21434, Invitrogen).

### Assay for Notch cis-inhibition and trans repression

For cloning of the Notch reporter construct (4xRBP)-luciferase the following DNA fragment was synthesized: TGAAAGTTACTGTGGGAAAGAAAGTTTGGGAAGTTTCACACGAGCCGTTCGCGTGCAGTCCCAGATATATATAGAGGCCGCCAGGGCCTGCGGATCACACAGGATCTGGAGCTGGTG. Using flanking restriction sites the synthesized fragment was cloned four times into the pGa981-6 vector [70], 5‘ to the β-globin minimal promoter followed by the firefly luciferase gene and the SV40 polyadenylation signal. To measure Notch cis-inhibition HeLa cells expressing endogenous Notch1 and Notch2 [52] were transiently transfected with 4 μg (4xRBP-Jκ)-luciferase, 0.5 μg Renilla luciferase and 1.5 μg DLL3, DLL3-S286A,T403V, or empty vector, respectively using Perfectin (Peqlab Biotechnology GmbH) according to the manufacturer‘s instructions. For Notch trans repression measurement CHO signal sending cells stably expressing DLL1 and DLL3 or mutant DLL3 were transfected with 1.5 μg Notch1, 1.5 μg LFNG, or empty expression vector. HeLa as signal receiving cells were transiently transfected with 4 μg (4xRBP-Jκ)-luciferase, 0.5 μg Renilla luciferase and 1.5 μg LFNG. 0.5 x 10^6^ transfected HeLa cells were cocultivated for 24 h on a 6-well plate with 0.5 x 10^6^ wild type CHO cells, or with CHO cells stably expressing DLL1 in duplicates. Using the Dual-Luciferase Reporter Assay System (Promega) luciferase activity was measured. Firefly luciferase activity was normalized to cotransfected Renilla-luciferase activity (pRL-TK, Promega) and to the expression levels of transfected proteins, which were verified by Western blot analysis and normalized to β-Actin.

### Metabolic labeling

CHO cells stably expressing DLL3 or DLL3 variants with mutated O-fucosylation sites were cultured in DMEM/F12 1:1, 10% FCS, Pen/Strep (Gibco) and GlutaMax (Gibco) containing 22 μCi/ml tritiated Fucose (Fucose, L-[5,6-^3^H], #ART0106A, American Radiolabeld Chemicals, Inc.) or 22 μCi/ml Met-[^35^S] (#IS-103, Hartmann Analytic). After 24 hours cells were used for immunoprecipitation as described earlier. Precipitated proteins were eluted from beads with 2 x sample buffer and separated on SDS-PAGE. Gels were stained for 15 min in Coomassie staining solution (40% Methanol, 0,04% Coomassie Brilliant blue R250, 20% Acetic acid), fixed for 15 min in fixation solution (5% Methanol, 7.5% Acetic acid), treated with Amplify Fluorographic Reagent (#NAMP100, Amersham) for 30 min, dried and labeled proteins were detected by autoradiography.

### Detection of sialic acid incorporation with "click" chemistry

CHO cells stably expressing LFNGHA were transfected with DLL3 expression constructs, treated with 100 μM Ac_4_ManNaz (tetraacetylated N-azidoacetyl-D-mannosamine, Click-iTManNAz metabolic glycoprotein labeling reagent, #C33366, Invitrogen/Molecular probes) in medium over night, and used for immunoprecipitation as described earlier. Beads with bound protein were washed three times in IP washing buffer without BSA. As a negative control proteins of one half of the beads were eluted in 2 x sample buffer. The other half of the beads was used for chemoselective ligation ("click" reaction) of alkyne-tagged Biotin (Click-iT detection reagent PEG4 carboxamide-propargyl biotin, #B10185, Invitrogen/Molecular probes) and the Click-iTProtein reaction buffer kit (#C10276, Invitrogen/Molecular probes) according to the manufacturer's instructions. "Click" modified proteins on beads were washed three times in IP washing buffer and eluted in 2 x sample buffer. After SDS-PAGE Biotin detection was performed by Western blot analysis with Streptavidin-HRP conjugate (#NEL750001EA, Perkin Elmer) or anti-Biotin antibody (Roche, 1426311), respectively.

### Generation of ES cells and single copy gene integration at the HPRT locus

Homozygous *Dll3* ^*pudgy*^, E14TG2a ES cell lines were obtained by cultivation of d4,5 blastocysts obtained from matings of double heterozygous E14TG2a, *Dll3* ^*pudgy*^ mice as described [71]. Transgene constructs to be expressed in the mouse embryo were introduced via SfiI into a modified version of the HPRT targeting vector pMP8 [57], containing the human HPRT promoter region including exon 1 in the 3‘ homology region (see Fig 5A). After homologous recombination of the vector into ES cells carrying a deletion in the HPRT gene (E14TG2a), HPRT function is restored and the resulting HAT resistance allows for the identification of correctly targeted ES cell clones. The mesoderm-specific promoter 2 (MSD2) used for gene expression consists of two copies of the MSD promoter previously described [59]. MSD2 was fused with a 780 bp fragment containing the minimal promoter and the first exon of *Dll1*. A KpnI site at the ATG start codon in *Dll1* was used to insert the LacZ-reporter, Flag-tagged *Dll3* or the Flag-tagged fucosylation mutant Dll3-S286A, T403V in frame. 3' the bovine growth hormone polyadenylation signal (BGH polyA) was added. Electroporated cells were selected for HAT resistance and correct recombination at the 5' end was verified by long range PCR (primers TGGGTTTCAAGATGTTCTGTTG and ACTCCTTCTGCTCTTCTTCTTTG) resulting in a 4060 bp PCR product. In addition ES cells were genotyped for the presence of the *pudgy* allele and the respective transgenes (see Genotyping of mutant embryos).

### Genotyping of embryos obtained from tetraploid complementation

Embryos obtained by injection of ES cells into tetraploid wild type embryos [72] were genotyped with respect to the *Dll3* transgenes by PCR and diagnostic restriction digests as follows (see Fig 5A): Primer pair TGTGAAGAGCCTGATGAATGC and TGGATGACCAAGAGTGCGG generates a 851 bp product specific for the transgene (*Dll3* cDNA), which is cleaved by Nar I in the presence of the S286A mutation into a 681 bp and 170 bp fragment (see Fig 5D). The same PCR product treated with Ale I results in a 526 bp and 325 bp fragment when amplified from the *Dll3* wt transgene, but is not cleaved in the presence of the T403V mutation (see Fig 5D).

### β-galactosidase staining of embryos

Chimeric embryos obtained after blastocyst injection of ES cells carrying the MSD2::LacZ transgene were collected at day 9 and β-galactosidase activity was detected as described [59].

### Immunocytochemistry

Immunocytochemistry was performed as described [37] and visualized using a laser scanning microscope (Leica DM IRB with a TCS SP2 AOBS scanhead). Pictures were processed using Leica Application Suite advanced fluorescence software (Leica AF) and Adobe Photoshop.

### Cycloheximide treatment

CHO cells stably coexpressing HA-tagged LFNG and Flag-tagged DLL3 or Flag-tagged DLL3-S286A,T403V, were treated with 100 μg/ml Cycloheximide and cell lysates were analyzed at different time points by Western blotting using the corresponding antibodies.

## Supporting Information

S1 Fig Variability of Uncx4.1 expression patterns in *DLL3* and *Lfng* mutants. Whole-mount in situ hybridizations of homozygous *Dll3* ^*pu*^ (a-c), homozygous *Lfng* ^*lacZ*^ (d-f) and double homozygous *Dll3* ^*pu*^; *Lfng* ^*lacZ*^ (g-i) E9.5 embryos using an Uncx4.1 probe. All three genotypes showed the same variations in the disturbance of A-P patterning of somites ranging from diffuse stripes to completely disorganized „salt-and-pepper”expression patterns (compare also with Fig 2b–2d).
(TIF)

S2 Fig Comparison of protein stability of wt and and mutant DLL3 in CHO cells. Western blot analysis of CHO cells stably expressing Flag-tagged wt or mutant DLL3 together with HA-tagged LFNG after cycloheximide treatment for different time periods indicated above revealed no obvious protein instability of DLL3-S286A,T403V compared with wt DLL3. The decrease of LFNG protein was used as a positive control for the successful treatment of the cells with cycloheximide. As a loading control a non-specific background band of the Flag antibody around 150 kDa was used.
(TIF)

S3 Fig Variability of *Uncx4.1* expression patterns in ES cell-derived *pudgy* and MSD2::*Dll3*-*S286A*, *T403V* transgenic embryos. Examples of whole-mount in situ hybridizations of completely ES cell derived embryos homozygous mutant for *Dll3* (*Dll3* ^*pu*^) and carrying the HPRT (ΔHPRT) deletion (a-c) and MSD2::*Dll3*-*S286A*, *T403V* transgenic embryos derived from two ES cell clones B5 (d-f) or B6 (g-i) showing a similar variable disorganized A-P pattern of the somites in both genotypes (compare also with Fig 5d, 5f and 5h).
(TIF)
