# Supplementary figures and images for: O-Fucosylation of DLL3 Is Required for Its Function during Somitogenesis
Source: PLoS One. 2015 Apr 9;10(4):e0123776. doi: 10.1371/journal.pone.0123776 (PMC4391858; doi:10.1371/journal.pone.0123776)

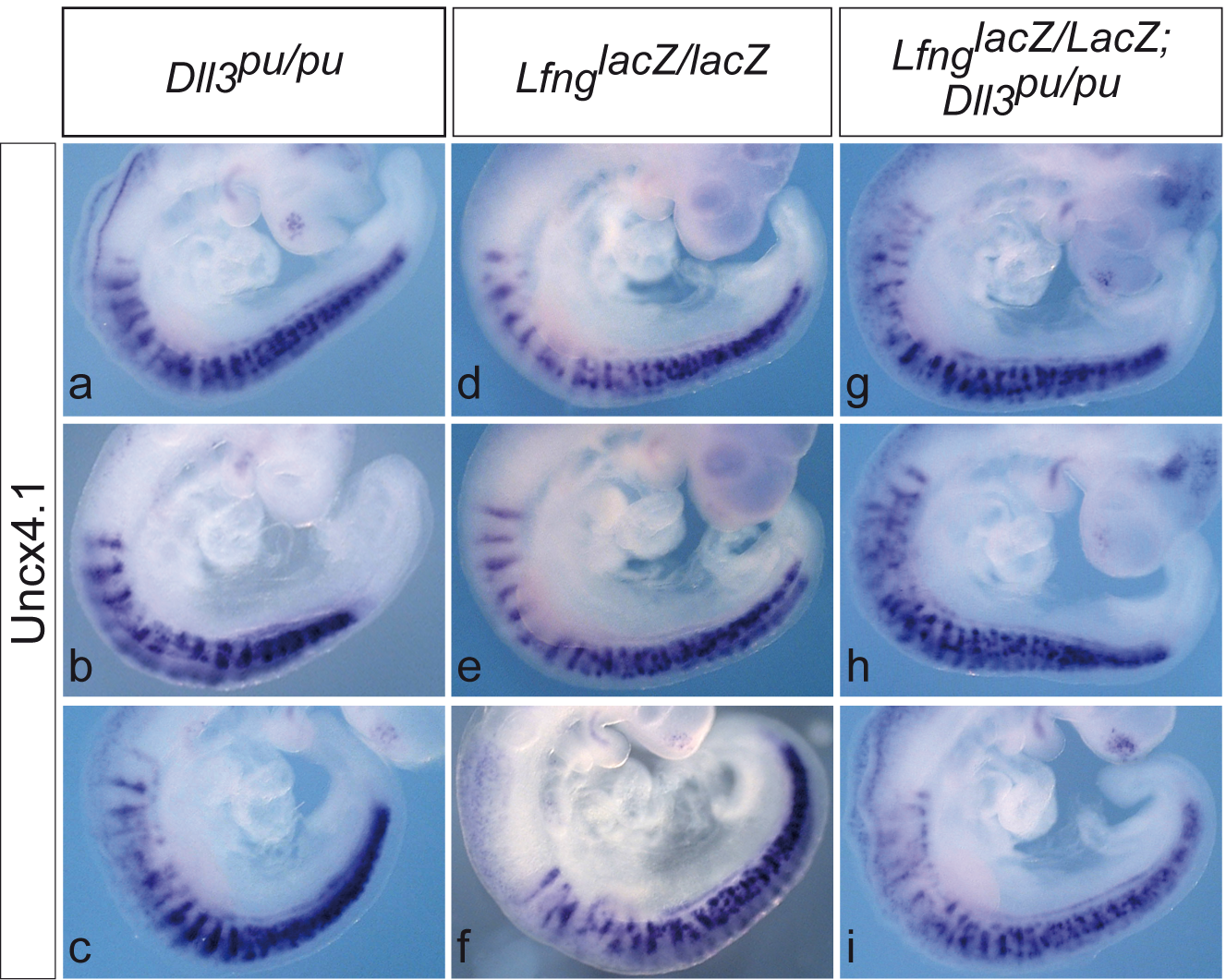

Supplement: S1 Fig — Whole-mount in situ hybridizations of homozygous Dll3 pu (a-c), homozygous Lfng lacZ (d-f) and double homozygous Dll3 pu; Lfng lacZ (g-i) E9.5 embryos using an Uncx4.1 probe. All three genotypes showed the same variations in the disturbance of A-P patterning of somites ranging from diffuse stripes to completely disorganized „salt-and-pepper”expression patterns (compare also with Fig 2b–2d). (TIF) [file pone.0123776.s001.tif]

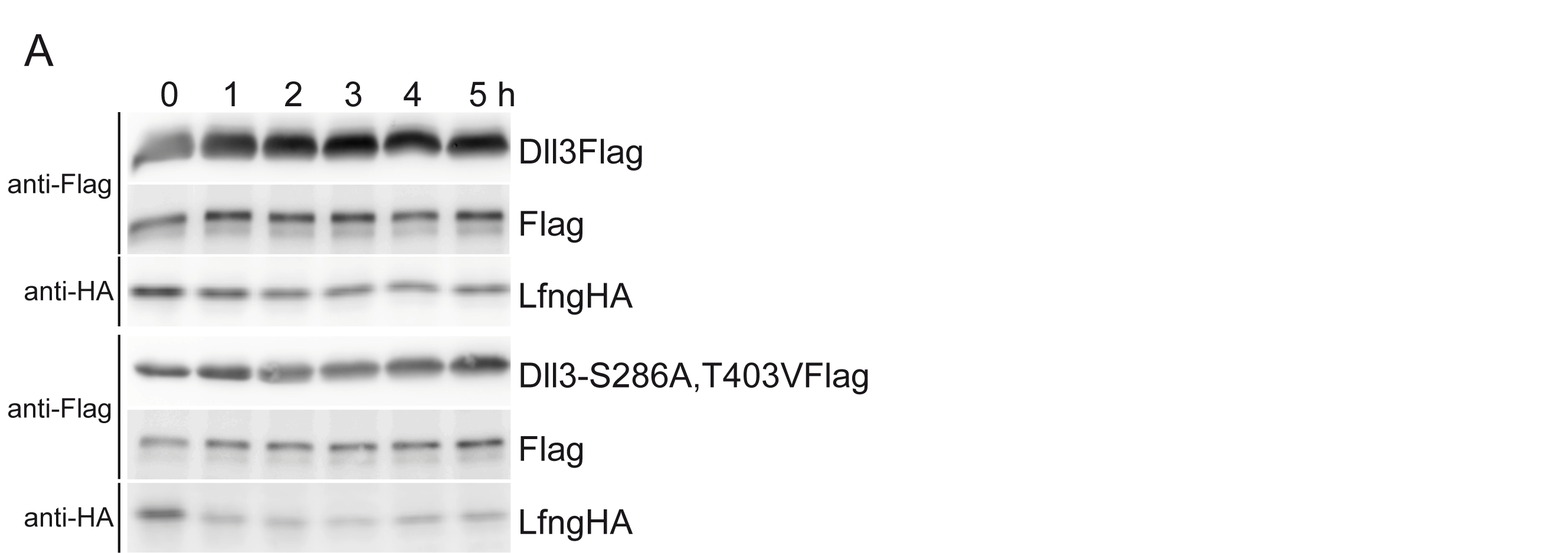

Supplement: S2 Fig — Western blot analysis of CHO cells stably expressing Flag-tagged wt or mutant DLL3 together with HA-tagged LFNG after cycloheximide treatment for different time periods indicated above revealed no obvious protein instability of DLL3-S286A,T403V compared with wt DLL3. The decrease of LFNG protein was used as a positive control for the successful treatment of the cells with cycloheximide. As a loading control a non-specific background band of the Flag antibody around 150 kDa was used. (TIF) [file pone.0123776.s002.tif]

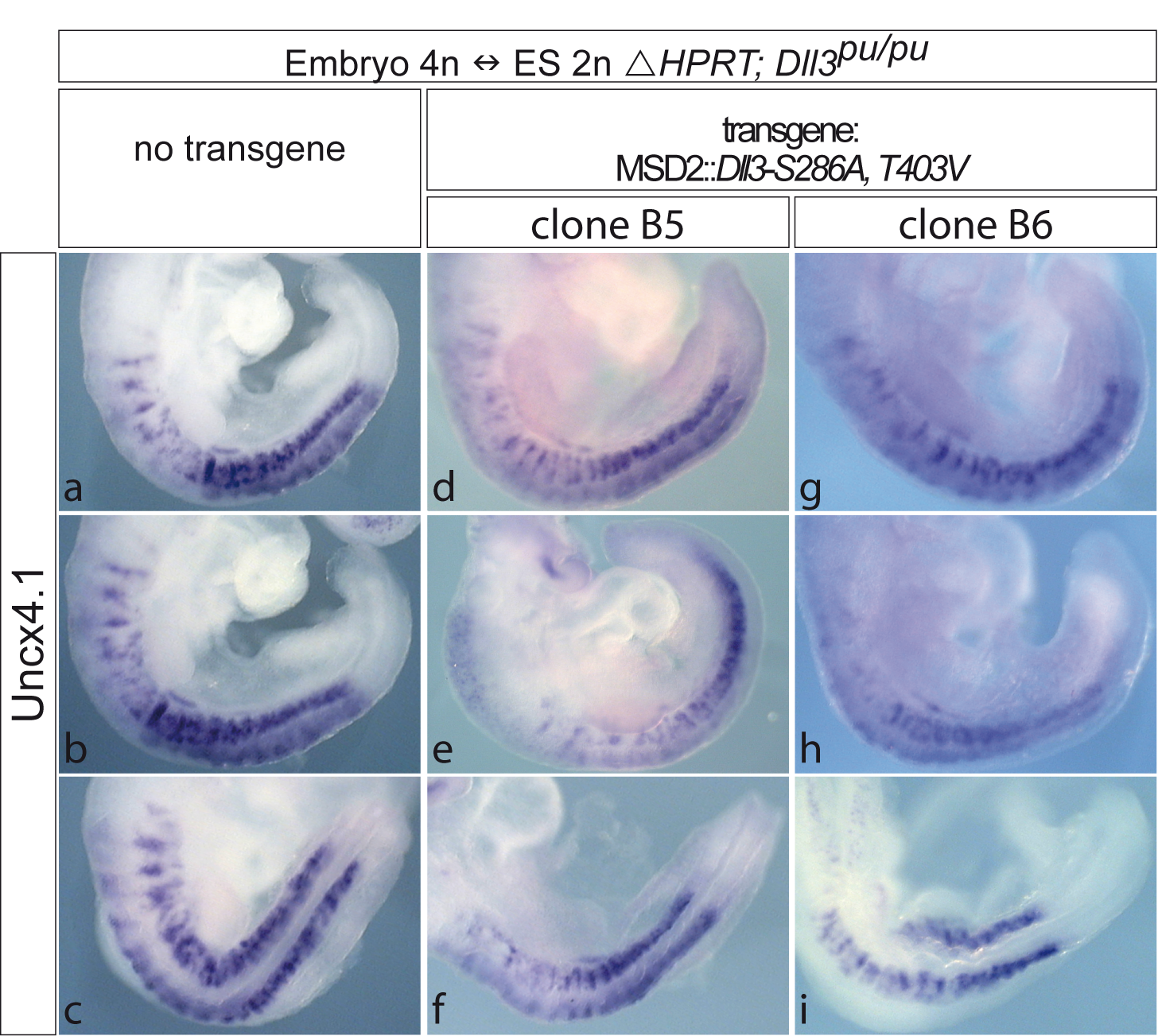

Supplement: S3 Fig — Examples of whole-mount in situ hybridizations of completely ES cell derived embryos homozygous mutant for Dll3 (Dll3 pu) and carrying the HPRT (ΔHPRT) deletion (a-c) and MSD2::Dll3-S286A, T403V transgenic embryos derived from two ES cell clones B5 (d-f) or B6 (g-i) showing a similar variable disorganized A-P pattern of the somites in both genotypes (compare also with Fig 5d, 5f and 5h). (TIF) [file pone.0123776.s003.tif]
